# Comment on “Correlation of L-asp Activity, Anti-L-asp Antibody, asn and gln with Adverse Events Especially Anaphylaxis Risks in PEG-asp-Contained Regime Treated Pediatric ALL”

**DOI:** 10.1177/15330338211049902

**Published:** 2021-11-05

**Authors:** Wing H. Tong

**Affiliations:** 1 Department of Public Health and Primary Care (PHEG), 4501 Leiden University Medical Center, Leiden, the Netherlands; 2 Argos Zorggroep “DrieMaasStede”, Center for Specialized Geriatric Care, Schiedam, the Netherlands

**Keywords:** pediatric acute lymphoblastic leukemia, asparaginase activity, asparagine, glutamine, anaphylaxis, asparaginase antibodies

## Dear Editor

I have read with great interest the publication by Wu *et al.*^1^ In the context of the Chinese Children's Cancer Group (CCCG)-ALL-2015 protocol, they studied the plasma L-asp activity/anti-L-asp antibody/asparagine/glutamine levels of 91 pediatric ALL patients who underwent PEG-asp-contained treatment on the seventh day after drug administration. Very recently, this CCCG-ALL-2015 protocol was used in an open-label, multicenter, randomized, phase 3, non-inferiority trial which involved twenty major medical centers across China.^2^ Yang *et al.* concluded that vincristine plus dexamethasone pulses might be omitted beyond one year of treatment for children with low-risk ALL.^2^ Wu *et al.* suggested that the measurement of L-asp activity/anti-L-asp antibody/asparagine/glutamine levels might assist the prevention of anaphylaxis-related AEs in pediatric ALL patients who underwent PEG-asp-contained treatment.^1^ Although this clinical study is of interest, some important questions can be raised, which I address below.

Wu *et al.* measured the PK/PD parameters during PEG-asp-contained treatment on day seven after administration. At that moment, no trough activity levels were monitored.^3^ Trough asparaginase activity levels of 100 U/L or greater appears to be a safe target level to ensure therapeutic benefit.^4^ Why did Wu *et al.* choose to use the peak levels on day 7 of PEGasparaginase?

Furthermore, anti-asparaginase antibodies and asparagine measurements are not indicated for clinical decision making outside the context of a clinical trial.^4^ Wu *et al.* studied anti-L-asp antibody, however, recent publications showed that not anti-L-asp antibody, but PEG is the major antigen that causes allergic reactions.^5^ Liu *et al.* concluded that anti-PEG-ASP has utility in predicting and confirming clinical reactions to PEGasparaginase as well as in identifying patients who are most likely to experience failure with rechallenge.^5^ Similar finding was found by the Dutch researchers, namely that anti-asparaginase antibodies were detected in only 11% during induction (of their DCOG ALL-11 protocol), but 94% during intensification.^6^ Wu *et al.* only studied the induction phase.^1^ Kloos *et al.* concluded, however, that anti-PEG and anti-SS-linker antibodies predominantly play a role in the immunogenic response to PEGasparaginase during induction.^6^ Of interest, these authors suggest that switching to native *Escherichia coli* asparaginase would be an option for adequate treatment. Do Wu and colleagues also have data on anti-PEG-ASP? If not, why did the authors only focus on anti-L-asp antibody?

Previously, it was published that no glutamine depletion was seen during PEGasparaginase therapy.^3^ Wu *et al.* suggested, however, that glutamine level might assist the prevention of anaphylaxis-related AEs.^1^ It is remarkable that these authors did find glutamine depletion, how could this phenomenon be explained? What was the role and the influence of day 7 post-infusion measurement on the glutamine level in this clinical study?

Finally, Wu *et al.* stated that for patients with plasma drug activity < 100 U/L, it was suggested to switch from PEGasparaginase to *Erwinia* asparaginase.^1^ These authors found that seven patients had anaphylaxis. These patients were switched to *Erwinia* asparaginase, were the data available after the switch on these parameters: L-asp activity/anti-L-asp antibody/asparagine/glutamine levels? This is particularly of interest as the number of patients switching to *Erwinia* asparaginase is currently limited.^7^

To conclude, monitoring of asparaginase PK by means of TDM is a powerful tool.^7^ This was confirmed by a recent expert panel discussion that agreed that TDM is useful to improve asparaginase efficacy.^8^ Wu *et al.* did a great job to study difficult PK/PD parameters in children suffering from ALL,^1^ however new PK/PD challenges need to be explored to further improve individualized treatments.

## Abbreviations

AE: adverse events
ALL: acute lymphoblastic leukemia
Anti-L-asp: anti-native *Escherichia coli* asparaginase
Anti-PEG-ASP: anti-polyethylene glycol asparaginase
Anti-SS-linker: anti-succinimidyl succinate linker
ASN: asparagine
DCOG: Dutch Childhood Oncology Group
*Erwinia* asparaginase: *Erwinia* chrysanthemi asparaginase
GLN: glutamine
L-asp: native *Escherichia coli* asparaginase
PEG: polyethylene glycol
PK/PD: pharmacokinetics/pharmacodynamics
TDM: therapeutic drug monitoring.

## References

[1] Wu J Chen C Huang S , et al. Correlation of L-asp activity, anti-L-asp antibody, Asn and Gln With adverse events especially anaphylaxis risks in PEG-asp-contained regime treated pediatric ALL patients. Technol Cancer Res Treat. 2020;19:1533033820980113. DOI: 10.1177/1533033820980113. 33287663 PMC7727045

[2] Yang W Cai J Shen S , et al. Pulse therapy with vincristine and dexamethasone for childhood acute lymphoblastic leukaemia (CCCG-ALL-2015): an open-label, multicentre, randomised, phase 3, non-inferiority trial. Clinical Trial Lancet Oncol. 2021; 22(9):1322–1332. DOI: 10.1016/s1470-2045(21)00328-4. 34329606 PMC8416799

[3] Tong WH Pieters R Kaspers GJ , et al. A prospective study on drug monitoring of PEGasparaginase and Erwinia asparaginase and asparaginase antibodies in pediatric acute lymphoblastic leukemia. Blood. 2014;123:2026-2033. DOI: 10.1182/blood-2013-10-534347. 24449211 PMC3968389

[4] van der Sluis IM Vrooman LM Pieters R , et al. Consensus expert recommendations for identification and management of asparaginase hypersensitivity and silent inactivation. Haematologica. 2016;101:279-285. DOI: 10.3324/haematol.2015.137380. 26928249 PMC4815719

[5] Liu Y Smith CA Panetta JC , et al. Antibodies predict pegaspargase allergic reactions and failure of rechallenge. J Clin Oncol. 2019;37:2051-2061. DOI: 10.1200/jco.18.02439. 31188727 PMC6804844

[6] Kloos R van der Sluis IM Mastrobattista E , et al. Acute lymphoblastic leukaemia patients treated with PEGasparaginase develop antibodies to PEG and the succinate linker. Br J Haematol. 2020;189:442-451. DOI: 10.1111/bjh.16254. 31883112

[7] Kloos RQH Pieters R Jumelet FMV , et al. Individualized asparaginase dosing in childhood acute lymphoblastic leukemia. J Clin Oncol. 2020;38:715-724. DOI: 10.1200/jco.19.02292. 31922920

[8] Baruchel A Brown P Rizzari C , et al. Increasing completion of asparaginase treatment in childhood acute lymphoblastic leukaemia (ALL): summary of an expert panel discussion. ESMO Open. 2020;5:e000977. DOI: 10.1136/esmoopen-2020-000977. 32967920 PMC7513670

